# Successful pancreatic pseudocyst drainage via the endoscopic minor papilla approach using a novel drill dilator

**DOI:** 10.1055/a-2740-3744

**Published:** 2025-12-08

**Authors:** Seiji Fujigaki, Tomoyuki Nakano, Yuta Sumida, Misaki Yokoi, Hideto Sugao, Tsuyoshi Sanuki

**Affiliations:** 1 610622 Department of Gastroenterology, Hyogo prefectural Harima-Himeji General Medical Center, Hyogo, Japan

Pancreatic pseudocysts are common complications of obstructive chronic pancreatitis. Endoscopic management includes transmural and transpapillary drainage, with the latter being appropriate when a pseudocyst communicates with the main pancreatic duct (MPD 1 ). When deep cannulation via the major papilla fails, access through the minor papilla may be necessary; however, minor papilla interventions are technically challenging owing to the narrow, tortuous course of the accessory pancreatic duct (APD) and the difficulty in maintaining endoscope stability. Recently, a novel drill dilator (Tornus ES; Olympus Medical Systems, Tokyo, Japan) has been reported to be effective for tract or stricture dilation in such challenging cases 2 3 .

A 75-year-old man presented with upper abdominal pain and vomiting. Computed tomography (CT) revealed acute pancreatitis with distal MPD dilation and a pancreatic head pseudocyst causing gastric outlet obstruction ( Fig. 1 ). The MPD stricture may elevate intraductal pressure, contributing to pseudocyst formation and pancreatitis. Therefore, transpapillary drainage was performed. Endoscopic retrograde pancreatography revealed a steep MPD preventing guidewire upstream passage. The initial guidewire was redirected into the APD and passed antegrade through the minor papilla into the duodenum. Minor papilla cannulation was achieved along the initial guidewire, and a second guidewire was introduced upstream of the MPD. Despite dilation using a tip-tapered bougie dilator and minor papilla sphincterotomy, device passage through the MPD stricture was unsuccessful. Subsequently, a drill dilator that could easily pass through and dilate the strictures was used. Pancreatography confirmed communication between the pseudocyst and the MPD, and a 5-Fr endoscopic nasopancreatic drainage (ENPD) tube was placed ( Fig. 2 ). No adverse events occurred, and follow-up CT revealed pseudocyst shrinkage ( Fig. 3 ). The ENPD tube was removed after pseudocyst resolution, and no recurrence was observed ( Video 1 ). Here, we reported the efficacy of a novel drill dilator in managing difficult strictures during minor papilla interventions.

**Fig. 1:**
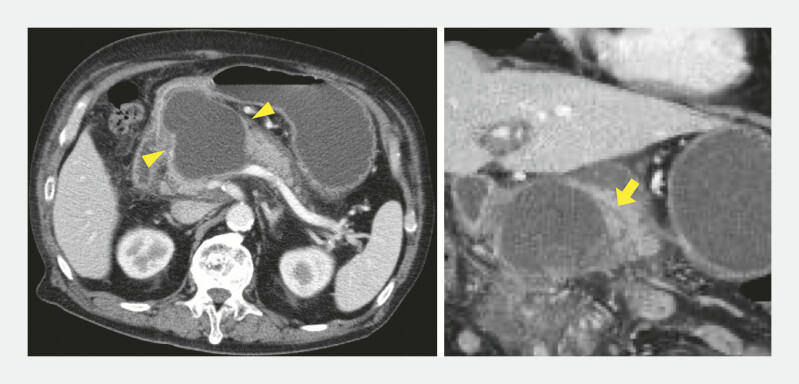
A computed tomographic image of the pancreatic pseudocyst (arrowhead) and the slightly dilated MPD (arrow). MPD, main pancreatic duct.

**Fig. 2:**
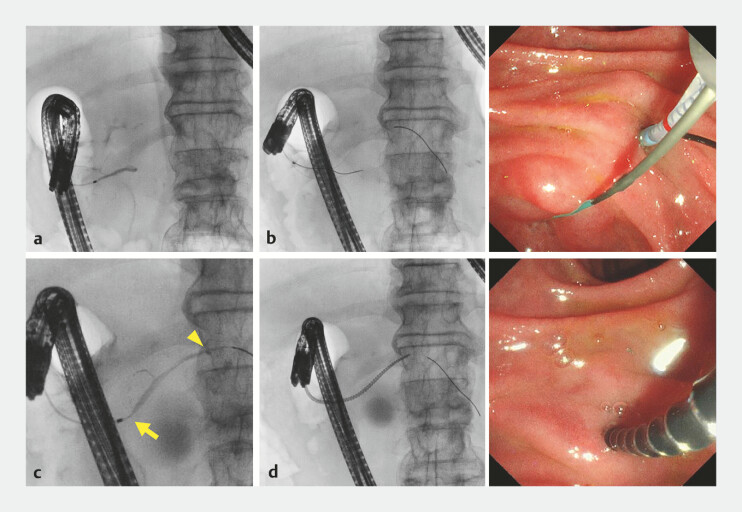
Endoscopic retrograde pancreatography performed to attempt transpapillary drainage. **a** Pancreatography demonstrating a steep shaped MPD. **b** Minor papilla cannulation is achieved along the initial guidewire, and a second guidewire is introduced upstream of the MPD. **c** A tight stricture is present in the MPD in the pancreatic head (arrow), and communication with the pseudocyst is identified in the pancreatic body (arrowhead). **d** The novel drill dilator can easily pass through and dilate the stricture. MPD, main pancreatic duct.

**Fig. 3:**
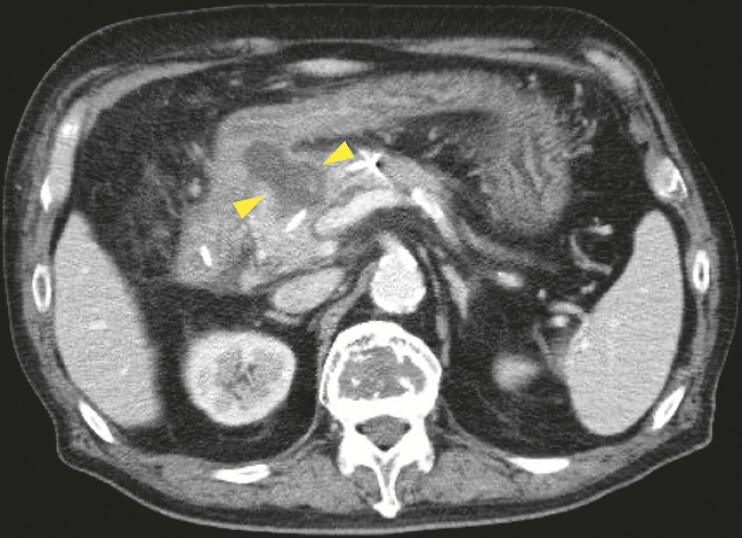
Follow-up CT demonstrating pseudocyst shrinkage (arrowhead). CT, computed tomography.

Endoscopic minor papilla intervention for a pancreatic pseudocyst using a novel drill dilator.Video 1

Endoscopy_UCTN_Code_TTT_1AS_2AJ

## Notes

Endoscopy E-Videos https://eref.thieme.de/e-videos
*E-Videos* is an open access online section of the journal *Endoscopy* , reporting on interesting cases and new techniques in gastroenterological endoscopy. All papers include a high-quality video and are published with a Creative Commons CC-BY license. Endoscopy E-Videos qualify for HINARI discounts and waivers and eligibility is automatically checked during the submission process. We grant 100% waivers to articles whose corresponding authors are based in Group A countries and 50% waivers to those who are based in Group B countries as classified by Research4Life (see: https://www.research4life.org/access/eligibility/ ). This section has its own submission website at https://mc.manuscriptcentral.com/e-videos .
